# *PpGRAS12* acts as a positive regulator of meristem formation in *Physcomitrium patens*

**DOI:** 10.1007/s11103-021-01125-z

**Published:** 2021-02-17

**Authors:** Hossein Beheshti, Christoph Strotbek, M. Asif Arif, Andreas Klingl, Oguz Top, Wolfgang Frank

**Affiliations:** 1 Plant Molecular Cell Biology, Department Biology I, LMU Biocenter, Ludwig-Maximilians-University Munich, Großhardener Straße 2-4, Planegg-Martinsried, Germany; 2 Plant Developmental Biology, Department Biology I, LMU Biocenter, Ludwig-Maximilians-University Munich, Großhardener Straße 2-4, Planegg-Martinsried, Germany

**Keywords:** GRAS family, CLAVATA, microRNA171, Growth arrest, Meristem regulation, Land plant evolution

## Abstract

**Key message:**

This study focused on the key regulatory function of *Physcomitrium patens GRAS12* gene underlying an increasing plant complexity, an important step in plant terrestrialization and the evolutionary history of life.

**Abstract:**

The miR171‐GRAS module has been identified as a key player in meristem maintenance in angiosperms. *PpGRAS12* is a member of the GRAS family and a validated target for miR171 in *Physcomitrium* (*Physcomitrella*) *patens*. Here we show a regulatory function of miR171 at the gametophytic vegetative growth stage and targeted deletion of the *PpGRAS12* gene adversely affects sporophyte production since fewer sporophytes were produced in Δ*PpGRAS12* knockout lines compared to wild type moss. Furthermore, highly specific and distinct growth arrests were observed in inducible *PpGRAS12* overexpression lines at the protonema stage. Prominent phenotypic aberrations including the formation of multiple apical meristems at the gametophytic vegetative stage in response to elevated *PpGRAS12* transcript levels were discovered via scanning electron microscopy. The production of multiple buds in the *PpGRAS12* overexpression lines similar to Δ*PpCLV1a/1b* disruption mutants is accompanied by an upregulation of *PpCLE* and downregulation of *PpCLV1, PpAPB, PpNOG1, PpDEK1, PpRPK2* suggesting that *PpGRAS12* acts upstream of these genes and negatively regulates the proposed pathway to specify simplex meristem formation. As CLV signaling pathway components are not present in the chlorophytic or charophytic algae and arose with the earliest land plants, we identified a key regulatory function of *PpGRAS12* underlying an increasing plant complexity, an important step in plant terrestrialization and the evolutionary history of life.

**Supplementary Information:**

The online version contains supplementary material available at 10.1007/s11103-021-01125-z.

## Introduction

The plant-specific *GRAS* genes encode transcriptional regulators that play key roles in plant growth and development. The name of GRAS derives from the first three GRAS proteins identified in *A. thaliana,* GIBBERELLIC ACID INSENSITIVE (GAI), REPRESSOR OF GA (RGA) and SCARECROW (SCR) (Di Laurenzio et al. 1996; Peng et al. 1997; Silverstone et al. 1998). Based on the protein sequence and structural characteristics, the GRAS protein family is divided into eleven subfamilies: DELLA, HAIRY MERISTEM (HAM), PHYTOCHROME A SIGNAL TRANSDUCTION1 (PAT1), LATERAL SUPPRESSOR (LAS) & SCARECROW-LIKE 4/7 (SCL4/7), SCARECROW (SCR), SHORT ROOT (SHR), SCARECROW-LIKE 3 (SCL3), LISCL (*L*l SCL), *Clonorchis sinensis* (*C. sinesis*) GRAS34 (CsGRAS34), *Oryza sativa* 19 (Os19) and DWARF AND LOW-TILLERING (DLT) (Zhang et al. 2019). GRAS protein subfamilies are known to be involved in various processes of plant growth and development such as gibberellin signal transduction (DELLA), radial root patterning and root growth (SCR and SHR), initiation and formation of axillary meristems (LAS), shoot meristem maintenance (HAM), phytochrome A signal transduction (PAT1 and SCL21), and gametogenesis (LlSCL) (Schumacher et al. 1999; Bolle et al. 2000; Helariutta et al. 2000; Wysocka-Diller et al. 2000; Greb et al. 2003; Morohashi et al. 2003; Engstrom 2012; Park et al. 2013; Torres-Galea et al. 2013). GRAS proteins also appeared to be involved in plant disease resistance and abiotic stress response (Mayrose et al. 2006).

The presence of a miR170/171 binding site within the encoded transcripts is characteristic for most members of the HAM family. MicroRNAs (miRNAs) are a class of non-coding ~ 21 nt RNAs that mediate gene silencing through cleavage of target messenger RNA (mRNA) and/or translational inhibition. miR171 is a conserved miRNA family that exists in all major land plant groups including bryophytes (Axtell and Bowman 2008) and plays critical roles in regulating plant growth and development through repressing expression of *SCARECROW-LIKE* (SCL) transcripts. In *P. patens*, two members of the *GRAS* family (Pp1s205_1V6.1/Pp3c12_10V3.1 and Pp1s130_63V6.1/ Pp3c7_1494V3.1) are validated targets of miR171 (Axtell et al. 2007; Hiss et al. 2017). Three members of the *HAM* subfamily in *A. thaliana* [*AtSCL6-II* (Atg45160), *AtSCL6-III* (At3g60630) and *AtSCL6-IV* (At4g00150) also known as the *HAM (HAIRY MERISTEM)* or *LOM (LOST MERISTEM)*] are reported targets of miR171 (Llave et al. 2002) and play an important role in shoot apical meristem maintenance and axillary meristem formation, polar organization and chlorophyll synthesis (Schulze et al. 2010; Wang et al. 2010). In *A. thaliana LOM1* and *LOM2* were shown to stimulate cell differentiation at the periphery of shoot meristems and to assist to maintain their polar organization (Schulze et al. 2010). Furthermore *AtHAM1, AtHAM2* and *AtHAM3* genes are not only essential for shoot apical meristem maintenance, but also play an important role in the maintenance of root indeterminacy (Engstrom et al. 2011). The *Petunia HAM* gene promotes shoot indeterminacy by an undefined non-cell-autonomous signaling mechanism (Engstrom et al. 2011). Tomato (*Solanum lycopersicum)* encodes three *HAM* homologs which are guided for cleavage by miR171 (Hendelman et al. 2016) and their silencing led to over-proliferation of cells in the periphery of the meristems. *SlHAM* genes not only function in meristem maintenance, but also play minor roles in the morphogenesis of a simple leaf that is determinate in tomato (Hendelman et al. 2016).

Apical meristems are a built by a specialized group of cells that principally reside at the tips of roots and shoots. Maintenance and programming of the meristematic cells are crucial steps for the cell division, shoot, and root branching. Any misregulation of the meristematic cells may result in perturbation and disorder in cell division, shoot, and root branching. Both shoot and root meristems are generated during embryogenesis, but do not contribute to the construction of the embryo and are activated once the seedling germinates (Doerner 2003). Following germination, the plant undergoes several developmental phases and shoot meristems change their identity in the course of these phase changes. In contrast, no identity alterations occur in root meristems during development. In *A. thaliana* the shoot meristem identity alteration appears as leaves during the initial vegetative growth, leaves and axillary meristems during the transition to flowering, and floral meristems and bracts by the inflorescence meristem during reproductive growth (Doerner 2003). Shoot apical meristems (SAMs) are responsible for developing the above-ground parts of the plant, such as stems, leaves, and flowers, while the under-ground parts of plants including root systems are generated by root apical meristems (Barton and Poethig 1993). The shoot apical meristem comprises a small bank of densely cytoplasmic, undifferentiated, dividing cells (Barton and Poethig 1993). Based on striking features including the ability of proliferation, regeneration into a new meristem after damage and the aptitude to produce a variety of differentiated cell types, meristem cells can be classified as stem cells (Sussex 1952; Potten and Loeffler 1990). Eight types of stem cells were reported to be formed in *P. patens* during its life cycle (Kofuji and Hasebe 2014). The common ancestor of land plants was haplontic and generated stem cells only in the gametophytic generation. Other types of body fragments in moss, such as the protonema and rhizoid filaments, leafy-shoot and thalloid gametophores, and gametangia were formed during land plant evolution by the divergence of stem cells in the gametophytic generation. Stem cells follow different morphological and anatomical patterns among land plants. The subapical cells of caulonemal filaments branch to form more filaments and three‐faced buds, which develop into leafy stems, called gametophores (Cove et al. 2009). While stem cells in shoot and roots of angiosperms and gymnosperms are multiple cells, in *P. patens* (protonema, gametophore, leaf, rhizoid, and sporophyte), stem cells are a single-cell (Kofuji and Hasebe 2014).

In plants, several transcription factors (TFs) were shown to be involved in meristem maintenance. Recessive mutations in the *WUSCHEL* (*WUS*) gene lead to an interruption in *A. thaliana* shoot meristem maintenance (Laux et al. 1996). The defect is restricted to shoot and floral meristems and persists at all developmental stages. In *A. thaliana, KANADI1* (*KAN1*), *KANADI2* (*KAN2*), *ASYMMETRIC LEAVES 2* (*AS2*), and *YABBY3* (*YAB3*) encode differentiation promoting TFs. WUS regulates *KAN1*, *KAN2*, *AS2*, and *YAB3* genes via direct binding to their regulatory regions to represses their expression (Yadav et al. 2013).

Another important signaling pathway involving CLAVATA (CLV) and WUS that controls stem cell maintenance via an auto-regulatory negative-feedback loop was first reported in *A. thaliana* (Schoof et al. 2000). WUS initially acts as an activator of *CLV3*, which further binds with the receptor kinase CLV1 and the receptor-like protein CLV2/CORYNE (CRN) complex and negatively regulates its own expression. The *A. thaliana jabba-1D* (*jba-1D*) mutant was reported to show multiple enlarged shoot meristems (Williams et al. 2005). Furthermore, *jba-1D* exhibits radicalized leaves, reduced gynoecia, and vascular defects. High *WUS* expression levels are detected in mutants since the *jba*-*1D* meristem phenotypes require a dramatic increase in *WUS* expression levels. Furthermore, overexpression of miR166g, which targets and reduces *PHABULOSA*, *PHAVOLUTA* and *CORONA* (*CNA*)/*ATHB15* transcript levels, is essential for the development of *jba-1D* meristem phenotypes*.* Williams et al. (2005) described the indirect involvement of miRNAs in controlling meristem formation via regulation of *WUS* expression. In addition to the WUS-CLV pathway, the ERECTA pathway, a leucine rich repeat receptor‐like kinase signaling pathway, represents an independent route that controls inflorescence architecture by regulating *WUS* expression (Mandel et al. 2014). Mutations of the eukaryotic translation initiation factor 3 subunit H, *eIF3h,* resulted in the formation of an enlarged shoot apical meristem in *A. thaliana* (Zhou et al. 2014). Unlike *A. thaliana*, homologs of CLV2 and CRN are missing in *P. patens*, but CLV3-like (CLE) peptides activate an intracellular MAPK signaling cascade through membrane localized CLV1 or RPK2 receptor kinases (Whitewoods et al. 2018). *P. patens* harbors two *CLV1* genes (*PpCLV1a-b)*, a single gene encoding an RPK2 receptor kinase (*PpRPK2*), one *NO GAMETOPHORES 1* gene (*PpNOG1*), four *AINTEGUMENTA, PLETHORA and BABY BOOM* genes (*PpAPB1-4*), one *DEFECTIVE KERNEL 1* (*PpDEK1*) and seven *CLE* (*PpCLE1-*7) genes and disruption of any of these genes adversely affects 2D to 3D transition in moss (Perroud et al. 2014; Demko et al. 2014; Johansen et al. 2016; Moody et al. 2018; Whitewoods et al. 2018). Analyses in *PpcleAmiR1-3*, *PpcleAmiR4-7*, Δ*Pprpk2*, Δ*PpDEK1*and Δ*PpCLV1a/1b* mutants showed division plane misorientation and/or defects in gametophore cell division and all of these mutant lines generated multiple/supernumerary buds, and hence defects in gametophore development (Perroud et al. 2014; Demko et al. 2014; Johansen et al. 2016; Whitewoods et al. 2018; Moody 2019).

Even though miR171 and its targets are known to play an important role in plant development and growth and are conserved within all major land plants, its regulatory functions in bryophytes have not been studied yet. In this study we show that *PpGRAS12* (Pp1s205_1V6.1/Pp3c12_10V3.1), a member of the GRAS family and a validated target for miR171 plays an important role in simplex meristem regulation. We provide evidence that PpGRAS12 negatively regulates *PpCLV1a-b, PpAPBs, PpNOG1, PpDEK1, PpRPK2* expression and causes the formation of multiple/supernumerary buds at the gametophytic vegetative stage in *P. patens.*

## Material and methods

### Plant material and growth conditions

All experiments were performed with *Physcomitrium* (*Physcomitrella*) *patens ssp. patens* (Hedwig) ecotype ‘Gransden 2004’ cultured under standard growth conditions as described by Reski and Abel (1985).

### Phenotypic analysis

Phenotypic analysis was performed by adjusting protonema cultures to an equal density of 100 mg/L dry weight and 5 µL of the adjusted cultures were spotted onto standard solid medium {250 mg L^−1^ KH_2_PO_4_, 250 mg L^−1^ KCl, 250 mg L^−1^ MgSO_4_ × 7H_2_O, 1 g L^−1^ Ca(NO_3_)_2_ × 4H_2_O and 12.5 mg L FeSO_4_ × 7H_2_O, pH 5.8 with micro-elements (ME) [H_3_BO_3_, MnSO_4_, ZnSO_4_, KI, Na_2_MoO_4_ × 2H_2_O, CuSO_4_, Co(NO_3_)_2_] and 12 g L^−1^ purified agar; Oxoid, Thermo Scientific, Waltham, MA, USA} or solid medium supplemented with 2 µM ß-estradiol (Sigma-Aldrich, St. Louis, USA) that was used as inducing agent for the *PpGRAS12* overexpression lines (*PpGRAS12*-iOE). Moss was grown in a growth cabinet under long day conditions (16 h light:8 h dark photocycle, 22 °C) at 100 µmol photons m^−2^ s^−1^. For the analysis of phenotypic changes at the leafy gametophore stage the inducer was directly applied onto colonies from transgenic lines as well as WT controls. Pictures of plants were taken by a Nikon stereoscopic microscope (C-DSD230, Minato, Japan).

### Subcellular localization of PpGRAS12

The complete *PpGRAS12* coding sequence was amplified by PCR from genomic DNA with the primers PpGRAS12::C_F and PpGRAS12::C_R containing *Sal*I and *Bgl*II restriction sites (Table S1). The PCR product was digested with *Sal*I *and Bgl*II and cloned into the *Sal*I- and *Bgl*II-sites of a modified pMAV4 plasmid (Martin et al. 2009), where the GFP reporter gene was replaced with a citrine coding sequence. The citrine coding sequence was C-terminally fused in frame to the *PpGRAS12* coding sequence and confirmed by sequencing. The resulting construct was transiently transfected into *P. patens* protoplasts following standard procedures (Frank et al. 2005). Nuclei were stained by the addition of 2.5 mg/mL of 4′,6-diamidino-phenylindole (DAPI, Sigma-Aldrich, USA). Fluorescence microscopy was performed using an inverted Leica TCS SP5 confocal laser scanning microscope (Carl Zeiss, Germany). The excitation/emission wavelengths were 514 nm/520–620 nm for YFP, 358 nm/460–490 nm for DAPI and 633 nm/650–720 nm for chlorophyll. Images were processed and assembled by ImageJ.

### Generation of ∆*PpGRAS12* mutants

The ∆*PpGRAS12* knockout construct was assembled by three-template PCRs and cloned into pJet1.2. These fragments were the 958 bp *PpGRAS12-*5′UTR, a 2174 bp *aph4* selection cassette conferring resistance to hygromycin and a 904 bp *PpGRAS12-*3′UTR. Primers used to generate these fragments are listed in Supplementary Table 1. The final insert was amplified via three templates PCR and inserted into pJet1.2. The insert was released from the vector backbone by *Kpn*I and *Nco*I to generate a fragment with 5′ and 3′ homologous ends and transfected into *P. patens* protoplasts (Frank et al. 2005). Subsequently, transgenic lines were selected on solidified Knop-ME medium containing 25 mg/L hygromycin. 50 plants surviving the selection procedure were directly screened by PCR for the integration of the ∆*PpGRAS12* knockout construct into the nuclear DNA. Positive lines were further analyzed by RT-PCR to confirm loss of the *PpGRAS12* transcript and subsequently by Southern blot to identify lines that harbor a single insertion at the *PpGRAS12* locus and to exclude additional genomic integration sites.

### Generation of *PpGRAS12*-iOE mutants

**Fig. 1:**
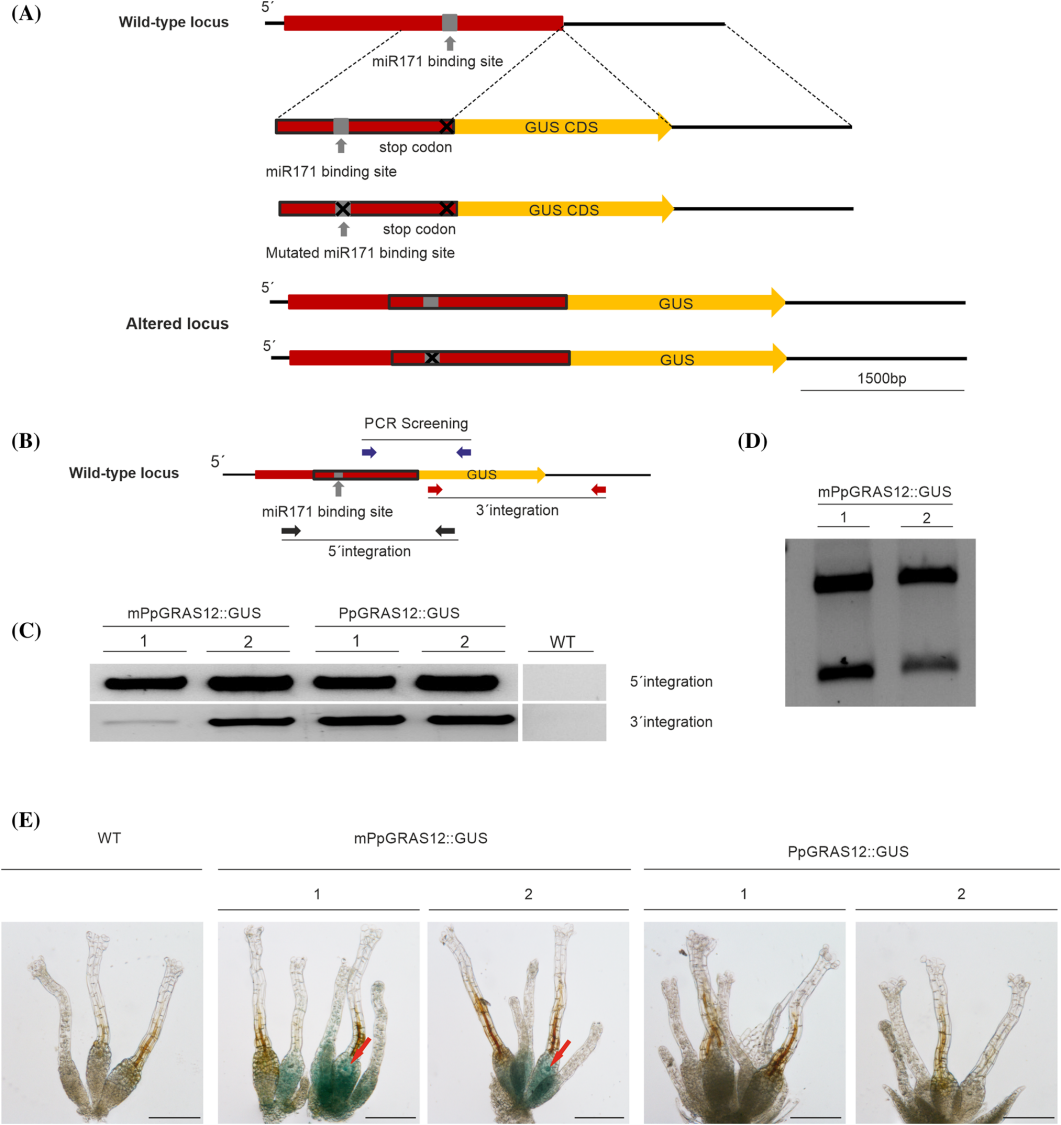
Generation of the PpGRAS12::GUS protein fusion reporter lines. **a** Scheme representing the generation of the *PpGRAS12*::*GUS* and m*PpGRAS12*::*GUS* fusion reporter constructs. Two variants of GUS fusion reporter constructs were generated and introduced to their cognate genomic locus by means of homologous recombination. The red box indicates the *PpGRAS12* coding region. The red box with the black border lines indicates 1482 bp from the coding sequence including the miR171 binding site (native/mutated), which was fused to the *GUS* coding sequence (yellow box). The *PpGRAS12* stop codon was removed and the coding sequence fused to the *GUS* coding sequence. **b** Purple, red, and black arrows show the primer pairs sequentially applied for PCR-based analyses of the PpGRAS12::GUS protein fusion reporter lines. **c** Upper panel: confirmation of 5′ integration of the constructs using black primers. Lower panel: confirmation of 3′ integration of the construct using red primers. **d** Validation of mPpGRAS12::GUS protein fusion reporter lines by digestion of RT-PCR products with *Pau*I (a *Pau*I restriction site was introduced into the mutated miR171 binding site). **e** Correspondent blue colors were detected only in the archegonia and egg cells of mPpGRAS12::GUS protein fusion reporter lines. Red arrows: egg cells. Scale bars: 1 mm

The complete *PpGRAS12* coding sequence harboring six neutral mutations within the miR171 binding site to prevent miR171-directed cleavage was amplified from the mutated version of *PpGRAS12* (Fig. 1). The cloning step was performed using the pENTR/D-TOPO cloning kits (Invitrogen, USA). A pair of primers (Supplementary Table 1) was designed to amplify the miR171-resistant *PpGRAS12* fragment from the plasmid harboring the mutated version of *PpGRAS12* and the amplified *PpGRAS12* fragment was cloned into the Gateway pENTR/D-TOPO vector (Invitrogen, USA). The fragment orientation was confirmed by sequencing and the pENTR/D-TOPO vector was cloned into the PpGX8 destination vector containing a hygromycin resistance cassette (Kubo et al. 2013) using the gateway LR Reaction (Invitrogen, USA). The inducible overexpression construct without homologous recombination sites was linearized using *Pme*I (NEB, USA) and transfected into *P. patens* protoplasts (Frank et al. 2005). Selection of stable transformants was performed as described above. 50 plants surviving the selection procedure were directly screened by PCR for integration of the *PpGRAS12* inducible overexpression construct into the nuclear DNA. Positive lines were further analyzed by RT-PCR and Northern blot to confirm an inducible overexpression of *PpGRAS12*.

### Generation of the PpGRAS12::GUS protein fusion reporter lines

The *PpGRAS12* coding sequence harboring a mutated or native miR171 binding site was fused to the GUS coding sequence and introduced to their cognate genomic locus by means of homologous recombination. Three sets of primers were used for the generation of PpGRAS12::GUS fusion constructs. The first set of primers was designed to amplify 1482 bp (5′ flanking region of the construct) from the coding sequence including the miR171 binding site, where the *Sac*I restriction site was added to the 5′ end and an *EcoR*I restriction site was added to the 3′ end. The second set including *EcoR*I and *Sal*I restriction sites was designed to amplify the *GUS* coding region. The third set of primers was designed to amplify 1528 bp downstream of the *PpGRAS12* coding region including the 3′ UTR (3′ flanking region of the construct), where the *Sal*I restriction site was added to the 5′ end and the *Kpn*I restriction site was added to the 3′ end. All three fragments were digested with *EcoR*I and *Sal*I, gel purified, ligated, and subsequently cloned into the pJET cloning vector. PpGRAS12::GUS and mPpGRAS12::GUS fusion reporter constructs were released from the pJET backbone by *Sac*I and *Kpn*I digestion and transfected into *P. patens* protoplasts. 50 plants surviving the selection procedure for each construct were screened by PCR to validate proper 5′ and 3′ integration of the GUS reporter constructs. PCR products from positive lines harboring the transfected constructs were further validated by sequencing.

### RNA isolation and qRT-PCR

Plant tissue was homogenized under liquid nitrogen and total RNA was extracted using TRI Reagent according to the manufacturer’s instructions (Sigma-Aldrich, St. Louis, USA). RNA was treated with RNase-free DNase I (NEB, USA), reverse transcribed as described Arif et al. (2018) and the synthesized cDNA was used as template for qRT-PCR analysis performed on a CFX96 Real-Time System (Bio-Rad, USA) using SYBR Green mix. The relative expression levels of genes were calculated using the 2^−ΔΔCT^ method (Livak and Schmittgen 2001) using two constitutively expressed *PpEf1α* (Pp1s7_445V6.1/Pp3c2_6650V3.1) and *PpC45*, encoding 60S ribosomal protein L21 (Pp1s107_181V6.1/Pp3c13_2360V3.1) as internal controls for normalization. Oligonucleotides used for qRT-PCR analyses are listed in Supplementary Table 1.

### Southern blot

Determination of copy numbers of inserted DNA fragments by Southern blot was performed according to QIAGEN Bench Guide (Qiagen, Hilden, Germany). 2 µg of genomic DNA was digested with the indicated restriction enzymes, resolved with PerfectBlue Gel System Mini L Revolution (PEQLAB Biotechnology, Erlangen, Germany) and blotted to a positively charged nylon membrane (Amersham Hybond-N+, GE Healthcare UK Limited, Buckinghamshire, UK). Samples were fixed by UV-crosslinking and hybridized with a [α^32^P] dCTP radioactively labeled probe which was amplified from selection cassette by PCR (Supplementary Table 1).

### RNA gel blot analysis

RNA gel blot analysis using 20 µg of total RNA was performed as described (Khraiwesh et al. 2008). A 721 bp *PpGRAS12* coding sequence was amplified with specific primers and used as a probe (Supplementary Table 1).

### Scanning electron microscopy

Gametophores from *P. patens* WT and mutant lines were fixed with 2.5% glutaraldehyde in 75 mM cacodylate buffer containing 2 mM MgCl_2_. After 4 washing steps with pure buffer, post-fixation was carried out with 1% OsO_4_ for 90 min. Two washing steps with buffer were followed by washing three times with double-distilled water. After this, the samples were dehydrated in a graded acetone series and critical-point-dried. Finally, the samples were mounted on aluminum stubs and sputter-coated with platinum. Scanning electron microscopy was performed on a Hitachi S-4100 SEM (Hitachi, Japan) at acceleration voltages between 3 and 5 kV.

## Results

### MiR171 regulates *PpGRAS12* expression

The existence of the GRAS domain categorized PpGRAS12 as a member of the GRAS family (Supplementary Fig. 1). *PpGRAS12* is a validated target of miR171 (Axtell et al. 2007) and belongs to the HAM subfamily (Chen et al. 2019). Plant miRNAs frequently play a role in defining the spatiotemporal expression of their cognate target mRNAs and the miR171‐GRAS module has been described as a key player in meristem maintenance in *A. thaliana* (Huang et al. 2017). To study whether miR171 regulates the spatiotemporal expression of *PpGRAS12*, PpGRAS12::GUS protein fusion reporter lines were generated. To generate these lines, the *PpGRAS12* coding sequence harboring a mutated or native miR171 binding site was fused to the *GUS* coding sequence and introduced to the cognate genomic locus by means of homologous recombination (Fig. 1a). Integrated DNA constructs were detected via PCR screening (purple primers, Fig. 1b) and the precise integration of the *PpGRAS12*::*GUS* fusion construct into the genome was confirmed for two independent lines by 5′ (black primers, Fig. 1b) and 3′ (red primers, Fig. 1b) integration PCR (Fig. 1c) and subsequent sequencing of the amplified products. Validation of the transgenic lines that had integrated the m*PpGRAS12::GUS* (miR171-resistant) construct was performed for two positive lines by subsequent digestion of RT-PCR products with *Pau*I since a *Pau*I recognition site was embedded within the miR171 binding site to generate silent mutations that prevent miR171-mediated cleavage of the *PpGRAS12* mRNA (Fig. 1d).

Histochemical GUS staining was performed for both miR171-resistant and miR171-sensitive lines. Correspondent blue color indicating GUS activity and expression of the *PpGRAS12* gene was neither observed in the native PpGRAS12::GUS or in the mutated mPpGRAS12::GUS protein fusion reporter lines at the protonema and gametophore stage (Fig. 1e). However, the blue color was only detected in the archegonia and egg cells of mPpGRAS12::GUS protein fusion reporter lines (Fig. 1e) resembling previously determined high levels of native *PpGRAS12* expression in the early phase of sporophyte development (Physcomitrium eFP browser; Ortiz-Ramírez et al. 2016). Expression of GUS in archegonia and egg cells of the miR171-resistant lines indicates that miR171 significantly regulates expression of *PpGRAS12* in these cells.

### Loss of the nuclear-localized PpGRAS12 protein results in reduction of sporophyte production

**Fig. 2:**
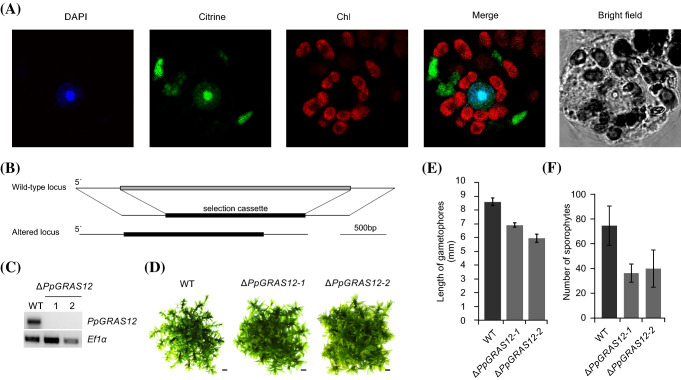
Generation and phenotypic analysis of the Δ*PpGRAS12* lines. **a** Subcellular localization of the PpGRAS12::citrine protein fusion in *P. patens* protoplasts. Pictures were taken 3 days after transfection of the *PpGRAS12*::*citrine* fusion construct into *P. patens* protoplasts. DAPI: DAPI signal. Citrine: citrine signal. Chl: chlorophyll auto-fluorescence. Merge: merged images of citrine and chlorophyll auto-fluorescence. **b** Scheme depicting the targeted knockout approach of the *PpGRAS12* coding sequence. **c** RT-PCR from cDNA derived from the indicated lines using *PpGRAS12*-specific primers; note that the two Δ*PpGRAS12* mutant lines are null mutants lacking the *PpGRAS12* transcript; RT-PCRs performed with primers for the constitutively expressed gene *PpEf1α* served as a control to monitor successful cDNA synthesis. **d** Phenotypic analyses of the knockout lines. Initially, a single gametophore from the indicated lines was cultured on standard growth medium and pictures were taken after 45 days of growth under standard growth conditions. Scale bars: 1 mm. **e** Comparison of the gametophore length in the WT and two independent ∆*PpGRAS12* lines. Gametophore length was measured from colonies grown for 45 days under standard growth conditions; error bars represent standard errors (n = 30). **f** Comparison of the sporophyte numbers in the WT and two independent ∆*PpGRAS12* lines; error bars represent standard errors (n = 27)

We performed transient expression of a C-terminal PpGRAS12-citrine protein fusion in *P. patens* protoplasts and confirmed an expected nuclear localization for the PpGRAS12 protein (Fig. 2a). The citrine fluorescence signals in the transformed protoplasts overlapped with nuclei stained by 4′,6-diamidino-2-phenylindole (DAPI), but we also observed signals in the cytosol. The observed nuclear localization was in agreement with the proposed function of GRAS proteins as transcription factors (Di Laurenzio et al. 1996; Gallagher and Benfey 2009; Heo et al. 2011; Yoshida et al. 2014). The cytoplasmic accumulation of PpGRAS12-citrine fusion protein might be due to a high ectopic expression level or the localization of PpGRAS12 into the nucleus may require the formation of heterodimers and a limited abundance of the dimerizing partner prevents complete targeting of PpGRAS12 to the nucleus and leads to remaining fluorescent signals in the cytoplasm.

To analyze the function of *PpGRAS12*, we generated ∆*PpGRAS12* knockout lines by targeted disruption of the corresponding genomic locus via the insertion of an *nptII* cassette (Fig. 2b). Using homologous recombination, gene targeting was performed and putative gene deletion mutants were first selected by 5′ integration PCR. Six out of 50 plants surviving the selection procedure and that showed proper 5′ integration were further analyzed by RT-PCR to confirm the loss of *GRAS12* transcript. Subsequently, two independent knockout lines with a single integration of the gene deletion construct were selected by Southern blot (Supplementary Fig. 2) and were further confirmed by reverse transcriptase PCR (RT-PCR) to be *∆PpGRAS12* null mutants that lack the *PpGRAS12* transcript (Fig. 2c). In the primary phase of growth including protonema and budding stage, no distinct differences were observed in the *∆PpGRAS12* lines compared to the WT. Mild phenotypic deviations were observed in the Δ*PpGRAS12* lines at the gametophytic growth stage (Fig. 2d) and confirmed by statistical analyses of the gametophore length in the WT and two independent Δ*PpGRAS12* lines (Fig. 2e). Further phenotypic analysis revealed that the absence of the *PpGRAS12* gene significantly influences the sporophyte production and consequently, fewer sporophytes were produced in the knockout lines compared to the WT (Fig. 2f).

### *PpGRAS12* overexpression leads to the formation of multiple apical simplex meristems

We observed a mild phenotypic deviation in the Δ*PpGRAS12* lines at the gametophytic growth stage (Fig. 2e, f) and prominent phenotypic aberrations in the sporophytic generation (Fig. 2f). To analyze the impact on *P. patens* growth and development upon *PpGRAS12* overexpression, *PpGRAS12* inducible overexpression lines (*PpGRAS12*-iOE lines) were generated. For this, we amplified the *PpGRAS12* cDNA and introduced six silent mutations within the miR171 binding site to inhibit miR171-mediated cleavage without affecting the encoded amino acid sequence (Supplementary Fig. 3). The fragment orientation was checked by sequencing and the pENTR/D-TOPO vector was cloned into the PpGX8 destination vector (Kubo et al. 2013). This construct was used for the transfection of *P. patens* protoplasts. After the selection of regenerating protoplasts on hygromycin-containing medium, a PCR-based screening was performed to identify desired transgenic lines within 50 plants surviving the selection period. The PCR-based screen (Supplementary Table 1) of regenerating lines identified two independent *PpGRAS12* overexpression lines (*PpGRAS12*-iOE). To verify the inducible expression of *PpGRAS12*, protonema tissue from both independent *PpGRAS12*-iOE lines was treated for 4 h with 2 μM ß-estradiol that was used in all experiments to induce *PpGRAS12* expression. Whereas the untreated *PpGRAS12*-iOE lines had similar *PpGRAS12* transcript levels as the WT control we detected a strong induction of the *PpGRAS12* transgene in both *PpGRAS12*-iOE lines by RNA gel blot (Supplementary Fig. 4). The overexpression of *PpGRAS12* in both *PpGRAS12*-iOE lines was detected in protonema 2 h after the addition of ß-estradiol (Supplementary Fig. 4a). Time course analysis of both *PpGRAS12*-iOE lines confirmed inducible and increasing *PpGRAS12* expression over time (Supplementary Fig. 4b).

**Fig. 3:**
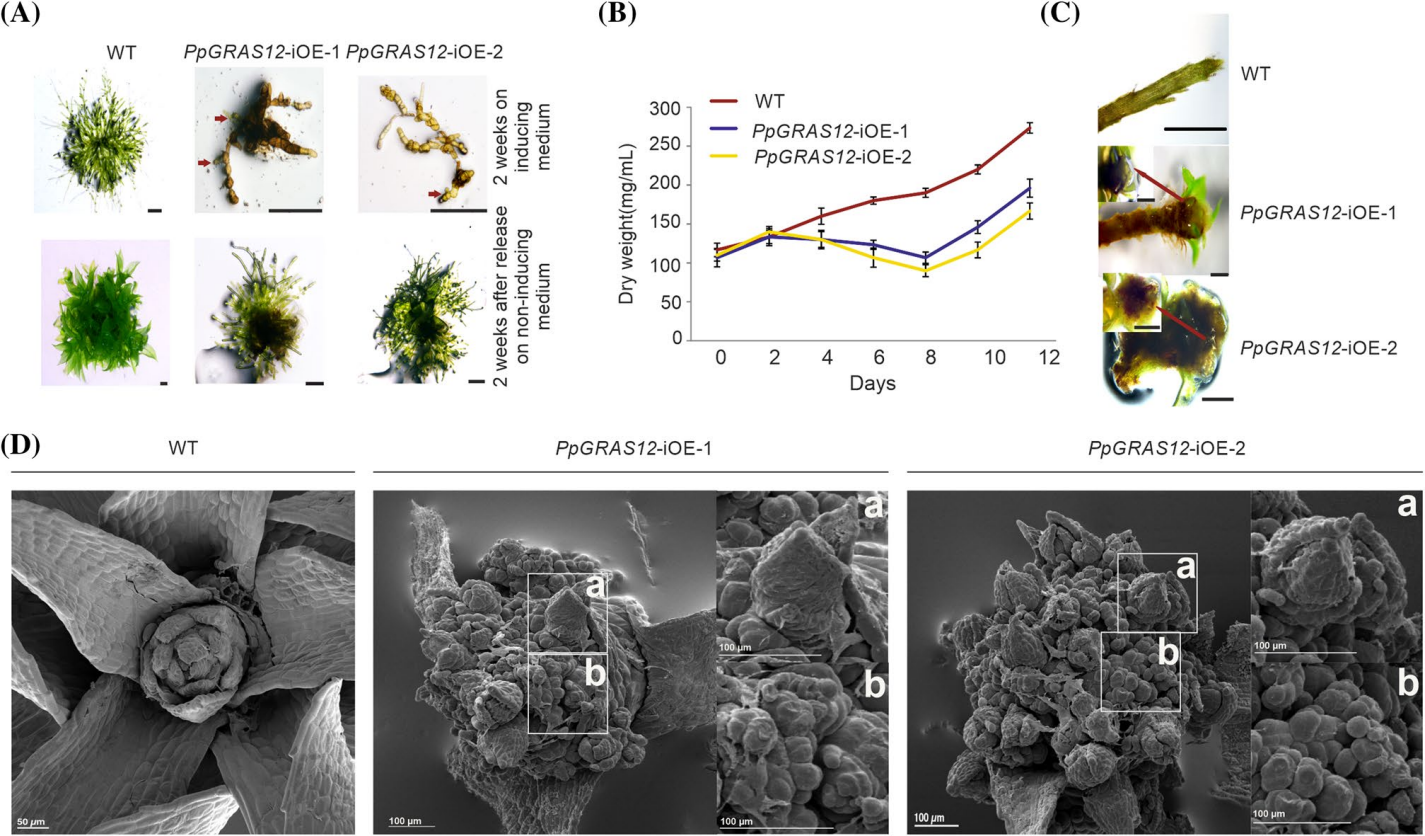
Phenotypic analysis of the *PpGRAS12*-iOE lines. **a** Equal amounts of protonema tissues from the WT and both *PpGRAS12*-iOE lines were spotted on standard solid growth medium supplemented with 2 µM ß-estradiol. Upper panel: protonema tissue after growth for 14 days on the medium supplemented with 2 µM ß-estradiol. Lower panel: 14 days after growth on inducing medium protonema tissue was transferred onto standard growth medium without inducer for 2 weeks. Red arrows indicate viable green cells. Scale bars: 1 mm. **b** *PpGRAS12*-iOE lines and WT were grown in standard liquid medium. Protonema from the *PpGRAS12*-iOE lines and WT were induced with 2 µM of ß-estradiol and dry weight of samples was measured every 2 days for a period of 12 days. Error bars indicate mean values ± SE (n = 3). **c** Formation of abnormal structures at the tip of both *PpGRAS12*-iOE lines. Scale bar: 1 mm for the WT and 0.5 mm for the mutants. **d** SEM analysis of *PpGRAS12*-iOE lines. The formation of supernumerary apical meristems in the *PpGRAS12*-iOE lines upon the induction with 2 µM of ß-estradiol. Box a: a leafy gametophore that was formed from an individual apical meristem. Box b: enlarged supernumerary apical meristems

Phenotypic analysis of the *PpGRAS12*-iOE lines in comparison to WT was performed by adjusting pure protonema cultures to an equal density of 100 mg/L dry weight and 5 µL of the adjusted cultures were spotted onto standard solid growth medium supplemented with 2 µM of ß-estradiol or without inducer. We did not observe any phenotypic differences between WT and both *PpGRAS12*-iOE lines on standard growth medium without inducer. However, highly specific and distinct growth arrests including a callus-like mass at the gametophore base and strong cell fate and proliferation defects were observed in the *PpGRAS12-*iOE lines upon the induction at the protonema stage (Fig. 3a, upper panel) whereas the *PpGRAS12*-iOE lines were able to recover and to return to normal growth and development after release to non-inducing conditions (Fig. 3a, lower panel). To analyze the growth behavior of the *PpGRAS12*-iOE lines in liquid medium, protonema tissue from WT and both *PpGRAS12*-iOE lines were transferred into liquid medium supplemented with 2 µM of ß-estradiol and growth of the cultures was monitored by the determination of the dry weight every 2 days. We observed a decrease in the growth rate of both *PpGRAS12*-iOE lines compared to the WT 2 days after the induction (Fig. 3b). The decrease in the growth rate of both *PpGRAS12*-iOE lines showed a steep decline until 8 days of growth in the induced medium followed by a slight recovery (Fig. 3c). However, the growth rate in both *PpGRAS12*-iOE lines remained lower compared to the WT after 12 days of growth in the induced medium. The slight increase after 8 days might be related to the gradual degradation of the inducer. Additionally, the effect of *PpGRAS12* induction at later growth stages was investigated using colonies that were grown on solid medium and developed leafy gametophores. For this, 2 µM of ß-estradiol was directly applied onto the colonies of both *PpGRAS12*-iOE lines as well as WT. Strikingly, atypical enlargement of the gametophore apical stem cells at the shoot apex was observed in both *PpGRAS12*-iOE lines 7 days after the induction (Fig. 3d). Furthermore, we noticed an abnormal enlarged structure at the tip of both *PpGRAS12*-iOE lines (Fig. 3d). Further investigation using scanning electron microscopy revealed that the abnormal structure, which was formed in response to an elevated level of *PpGRAS12* at the tip zone*,* is formed by multiple apical simplex meristems (Fig. 3d).

**Fig. 4:**
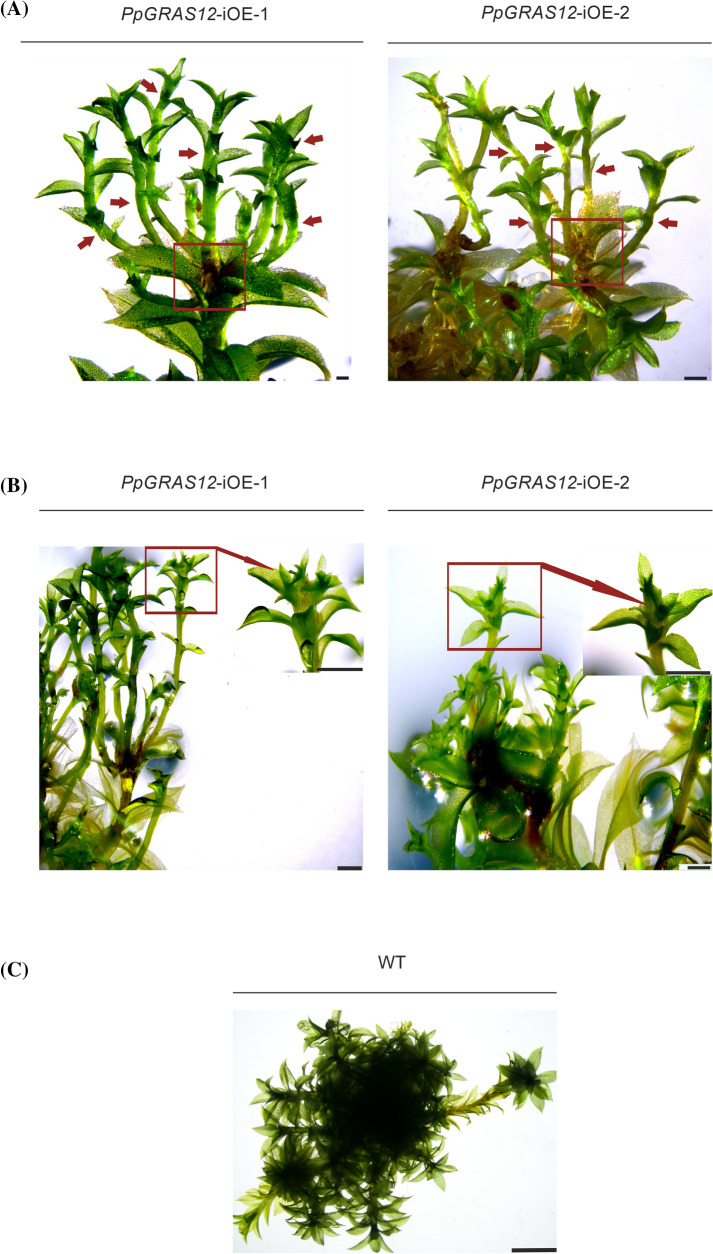
Supernumerary gametophore formation from multiple apical meristems in the induced *PpGRAS12-*iOE lines. **a** Supernumerary gametophores were formed from multiple apical meristems in the *PpGRAS12*-iOE lines upon *PpGRAS12* induction. The red box shows the development of multiple gametophores from apical meristems in the *PpGRAS12*-iOE lines. Red arrows indicate a single gametophore. Pictures were taken 75 days after the induction; scale bar: 1 mm. **b** Renewal of induction resulted in the formation of multiple apical meristems and consequently, the formation of multiple gametophores on the top of previous gametophores. The red arrow shows multiple gametophores. Pictures were taken, 12 days after the renewal of induction; scale bar: 1 mm. **c** Representative WT gametophores grown on standard solid growth medium; scale bar: 100 µm

**Fig. 5:**
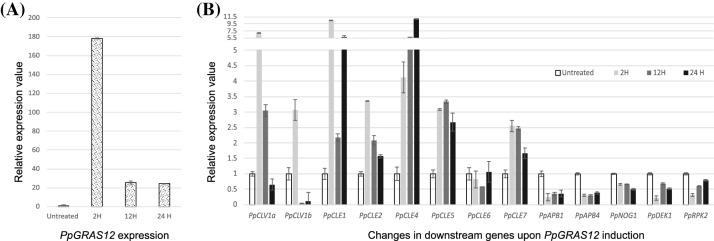
The relative expression levels of *PpGRAS12* and other members of MAPK signaling pathway in the WT and *PpGRAS12*-iOE lines. **a** The relative expression levels of *PpGRAS12* 2 h, 12 h and 24 h after induction. **b** The relative expression levels of *PpCLV1a, PpCLV1b, PpCLE1, PpCLE2, PpCLE4, PpCLE5, PpCLE6, PpCLE7, PpAPB1, PpAPB4, PpNOG1, PpDEK1 and PpRPK2* 2 h, 12 h and 24 h after induction of the *PpGRAS12* gene. Plants were grown on standard solid growth medium for 4 weeks, induced for 2 h, 12 h and 24 h and RNA from gametophore tissue was used for qRT-PCR. Relative gene expression levels were normalized to *PpEf1a* and *PpC45* and transcription rates in the uninduced *PpGRAS12*-iOE line were set to 1. Error bars indicate mean values ± SE (3 biological replicates with three technical replicates each). Statistically significant differences for each gene were analyzed by one-way ANOVA (p < 0.05)

Moreover, after withdrawal of the inducer for 3 weeks causing its dilution and/or degradation individual apical cells were able to undergo normal developmental progression and developed into leafy gametophores (Figs. 3d, 4a). However, the developmental progression of *PpGRAS12*-iOE lines was still significantly delayed compared to WT. Furthermore, if a new gametophore, which has previously emerged from an individual apical simplex meristem, once more was exposed to the inducer, multiple apical simplex meristems were formed over again from the tip (Fig. 4b). *A. thaliana CLV1* was previously reported to play an important role in maintaining meristem identity and controlling meristem size (Clark et al. 1993) since the *A. thaliana clv1* mutant develops enlarged shoot apical meristems (Clark et al. 1995; Shinohara and Matsubayashi 2015). To investigate whether the formation of multiple apical meristms upon *PpGRAS12* overexpression might be caused by reduced levels of the *P. patens CLV1a* and *CLV1b* homologs as well as altered expression levels of additional genes that act in the signaling cascade we analyzed their expression levels in one representative *PpGRAS12*-iOE line by qRT-PCR 2 h, 12 h and 24 h after the induction of *PpGRAS12* in gametophores. Since we did not detect differential expression of the analyzed genes between WT and the uninduced *PpGRAS12*-iOE line, the uninduced *PpGRAS12*-iOE line was used as a control. Even though an over 175-fold increase in the *PpGRAS12* expression level was observed 2 h after induction, the transcript levels dropped to an approximately 25-fold increase and remained constant after 24 h of induction (Fig. 5a). 24 h after induction, all *CLE* genes, *PpCLE1, PpCLE2, PpCLE4, PpCLE5, PpCLE6* and *PpCLE7*, were upregulated upon *PpGRAS12* induction (Fig. 5b). *PpCLV1* expression analysis showed downregulation of both *PpCLV1a* and *PpCLV1b* homologs in response to the elevated *PpGRAS12* levels (Fig. 5b). Statistically significant differences for each gene were analyzed by one-way ANOVA and other than *PpCLE6*, changes in relative expression levels were significantly different (p < 0.05). Furthermore, qRT-PCR analysis also revealed the downregulation of *PpAPB1, PpAPB4, PpNOG1, PpDEK1 and PpRPK2* upon *PpGRAS12* induction. The downregulation of both *PpCLV1a* and *PpCLV1b* as well as reduced transcript levels of additional genes acting downstream might explain the formation of multiple simplex meristems similar to Δ*PpCLV1a/1b*, *Pprpk2* and Δ*PpDEK1* disruption mutants*.*

## Discussion

PpGRAS12 is a member of the GRAS family (Pysh et al. 1999; Tian et al. 2004; Hirsch and Oldroyd 2009) harboring a conserved order of the characteristic GRAS motifs. Here, we show that PpGRAS12 is nuclear-localized, which is in agreement with the proposed function of GRAS proteins as transcription factors (Di Laurenzio et al. 1996; Gallagher and Benfey 2009; Heo et al. 2011).

The presence of the miR170/171 binding site is a characteristic feature for most members of the *HAM* families. *A. thaliana* orthologs of *Petunia HAM* were shown to be targets of miR170/171 (Llave et al. 2002) and they were shown to be involved in meristem regulation and the CLV-WUS pathway (Zhou et al. 2018) that controls stem cell maintenance via an auto-regulatory negative-feedback loop (Schoof et al. 2000). HAM and WUS share collective targets in vivo and their physical interaction is vital in driving downstream transcriptional programs and promoting shoot stem cell proliferation (Zhou et al. 2015). *AtGPR23*, *AtTIT2;2,* and *AtTPL* are reported as collective targets of HAM and WUS and they are noticeably affected when WUS and HAM interact (Zhou et al. 2015). Analysis of the PpGRAS12::GUS protein fusion reporter lines showed a regulatory function of miR171 in *PpGRAS12* expression. We observed a noticeable expression of the *PpGRAS12* gene in the archegonia and egg cells of the mPpGRAS12::GUS protein fusion reporter lines compared with the PpGRAS12::GUS lines and WT suggesting that miR171 controls the expression of *PpGRAS12* in *P. patens* archegonia and egg cells. Loss of function ∆*PpGRAS12* lines displayed a fewer number of sporophytes compared to the WT. Based on the elevated expression of *PpGRAS12* in the egg cells of the mPpGRAS12::GUS protein fusion reporter lines and reduced sporophyte production in the ∆*PpGRAS12* lines, we suggest that *PpGRAS12* plays a role in egg cell regulation and sporophyte production.

A callus-like mass at the gametophore base and strong cell fate and proliferation defects were observed in the *PpGRAS12-*iOE lines at the protonema stage upon the induction of *PpGRAS12* expression. Furthermore, we observed the formation of multiple apical simplex meristems at the tips of gametophores upon *PpGRAS12* induction in the *PpGRAS12*-iOE lines. The shoot apical meristem (SAM) is responsible for the post-embryonic growth and generates plant aerial structures. Widespread variations in shoot meristem structure have evolved among living plant lineages such as the single-celled apices of the non-vascular bryophytes, the multicellular meristems with prominent apical cells in the seedless lycophytes, and the multiple cell-layered shoot meristems of angiosperms. An appropriate continuous growth in plants depends on the SAM ability to maintain the balance between self-renewal of stem cells and cell recruitment for lateral organ formation (Lee et al. 2019). The WUS and CLV signaling pathways are key factors of meristematic activity in the SAM (Laux et al. 1996). The *A. thaliana clv1* mutant develops enlarged shoot apical meristems (Clark et al. 1995). Furthermore, mutation of the *CLV1* gene has resulted in an increased number of all floral organ types (Leyser and Furner 1992). On the other hand, disruption of any gene (*PpCLV1a-b*, *PpCLE1-7* and *PpRPK2*) involved in the MAPK signaling cascade which is responsible for 2D to 3D transition in *P. patens* resulted in the formation of supernumerary buds and defective gametophores*.* We observed the same phenomenon, the formation of enlarged and multiple apical simplex meristems in *PpGRAS12*-iOE lines in response to an elevated level of *PpGRAS12.* The qRT-PCR analyses revealed the upregulation of *PpCLE* genes and concomitant downregulation of *PpCLV1a/b, PpAPB, PpNOG1, PpDEK1 and PpRPK2* genes upon elevated levels of *PpGRAS12* indicating that *PpGRAS12* acts upstream of these genes and induces the formation of multiple and enlarged apical simplex meristems. A detailed analysis at earlier time points revealed that both *PpCLV1a* and *PpCLV1b* were initially upregulated and then downregulated. Despite the upregulation of *PpCLE* genes, the downregulation of genes encoding membrane-localized receptor kinases (*PpCLV1* and *PpRPK2*) led to downregulation of the MAPK signaling pathway and resulted in downregulation of *PpAPB* genes. Surprisingly, we could not observe an antagonistic interaction between *PpDEK1* and *PpNOG1* in controlling *PpAPB* genes, like in the previously proposed model for 3D growth regulation in *P. patens.* This difference might be due to the fact that the proposed model explains underlying molecular changes during the formation of gametophore initial cells from caulonemal filaments and the subsequent acquisition of a tetrahedral apical cell (Moody 2019). Hence, the antagonistic interaction between *PpDEK1* and *PpNOG1* in controlling *PpAPB* genes might only occur during the formation of gametophore initial cells, but not in multiple apical simplex meristem formation. Taken together, our results indicate the involvement and key role of *PpGRAS12* in simplex meristem regulation, maintenance and identity control and provides new insights into the MAPK signaling pathway*.* Further investigations are needed to elucidate how these genes and their feedback loops together with other players in the MAPK signaling pathway as well as cytokinin signaling function synergistically in the formation and development of gametophores. Consequently, this will help us to understand innovations related to an increased body plan complexity during the water-to-land transition of the earliest land plants.

## Supplementary Information

Below is the link to the electronic supplementary material.

Electronic supplementary material 1 (DOC 766 kb)
