# Supplementary material for: PpGRAS12 acts as a positive regulator of meristem formation in Physcomitrium patens
Source: Plant Mol Biol. 2021 Feb 17;107(4-5):293–305. doi: 10.1007/s11103-021-01125-z (PMC8648639; doi:10.1007/s11103-021-01125-z)
Supplement: Supplementary file 1 — Electronic supplementary material 1 (DOC 766 kb) [file 11103_2021_1125_MOESM1_ESM.doc]

# Supplementary Material

# *PpGRAS12* acts as a positive regulator of meristem formation in *Physcomitrium patens*

# Hossein Beheshti^1^, Christoph Strotbek^1^, M. Asif Arif^1^, Andreas Klingl^2^, Oguz Top^1^ and Wolfgang Frank^1,*^

# ^1^Ludwig-Maximilians- University Munich, Plant Molecular Cell Biology, Department Biology I, LMU Biocenter, Großhardener Straße 2-4, Planegg-Martinsried, Germany

^2^Ludwig-Maximilians- University Munich, Plant Developmental Biology, Department Biology I, LMU Biocenter, Großhardener Straße 2-4, Planegg-Martinsried, Germany

*Author for correspondence:

Wolfgang Frank

Tel: +49 (0)89 2180-74670

*Email:* [*wolfgang.frank@lmu.de*](mailto:wolfgang.frank@lmu.de)

The following Supporting Information is available for this article:

**Fig. S1** GRAS domain structure in PpGRAS12

**Fig. S2** Southern blot with an *aph*4 specific probe for the identification of single integration Δ*PpGRAS12* lines.

**Fig. S3** Silent mutations within the *PpGRAS12* miR171 binding site.

**Fig. S4** Validation of β-estradiol induced *PpGRAS12* expression in protonema tissues of *PpGRAS12*-iOE lines by Northern blots.

**Table S1** List of primers used for the experiments


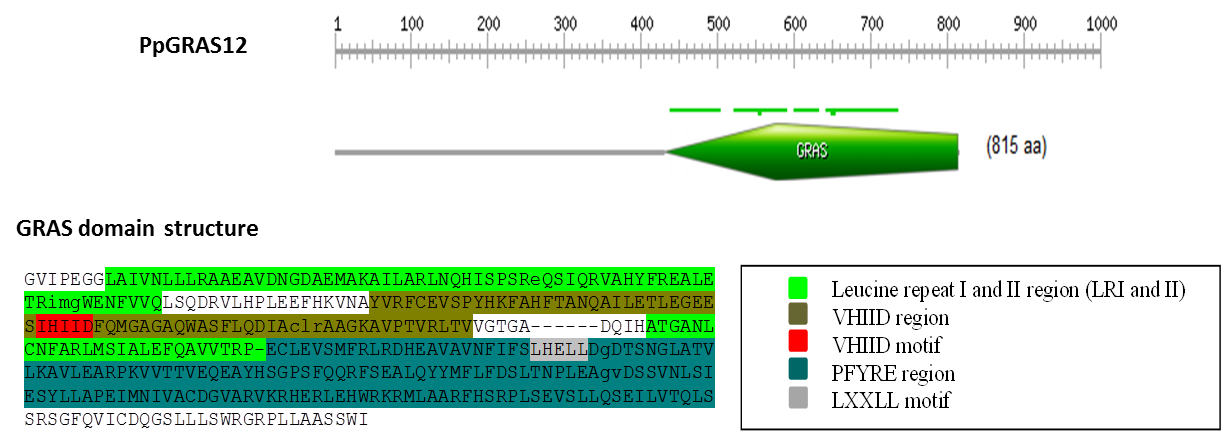


**Fig. S1** GRAS domain structure in PpGRAS12. GRAS domain prediction and GRAS domain motifs prediction using EXPASY-PROSITE [(https://prosite.expasy.org/).](../../../../Physco-staff/Hossein-Beheshti/GRAS%20project/GRAS%20paper/GRAS-Paper-edited/final/(https:/prosite.expasy.org/).)

[
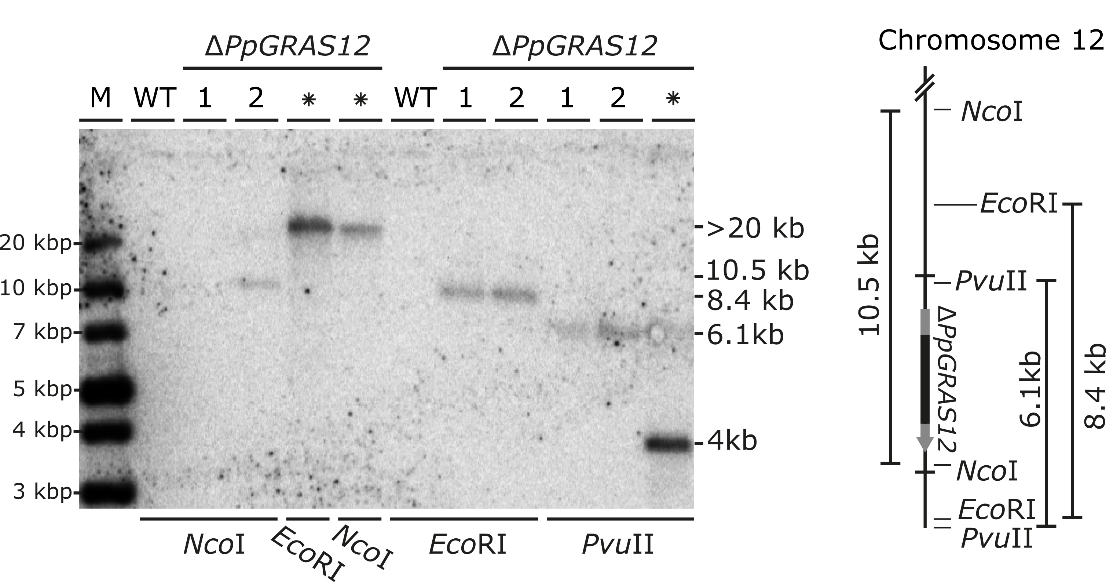
](../../../../Physco-staff/Hossein-Beheshti/GRAS%20project/GRAS%20paper/GRAS-Paper-edited/final/(https:/prosite.expasy.org/).)

**Fig. S2** Southern blot with an *aph*4 specific probe for the identification of single integration Δ*PpGRAS12* lines. 2 µg of digested gDNA were resolved. The signal of in Δ*PpGRAS12-1* gDNA digested by *Nco*I is presumably below the detection limit. Schematic representation of the Δ*PpGRAS12* locus on chromosome 12 and restriction enzymes´ positions as well as the resulting fragment sizes are shown on the right side. Lanes marked with asterisk are not single integration lines and were not further analyzed.


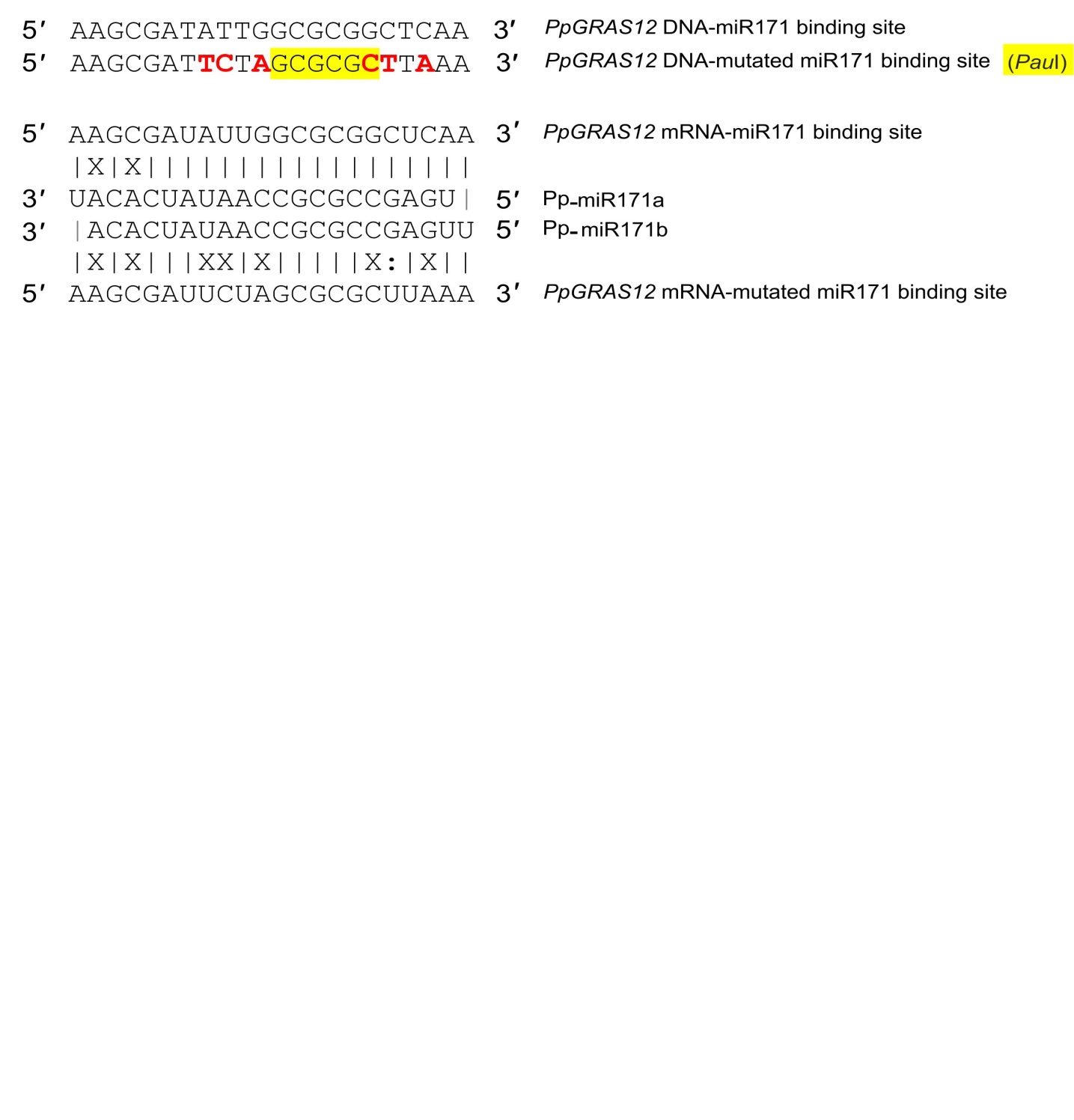


**Fig. S3** Silent mutations within the *PpGRAS12* miR171 binding site. Yellow box: restriction site for *Pau*I. Red nucleotides indicate silent mutations.


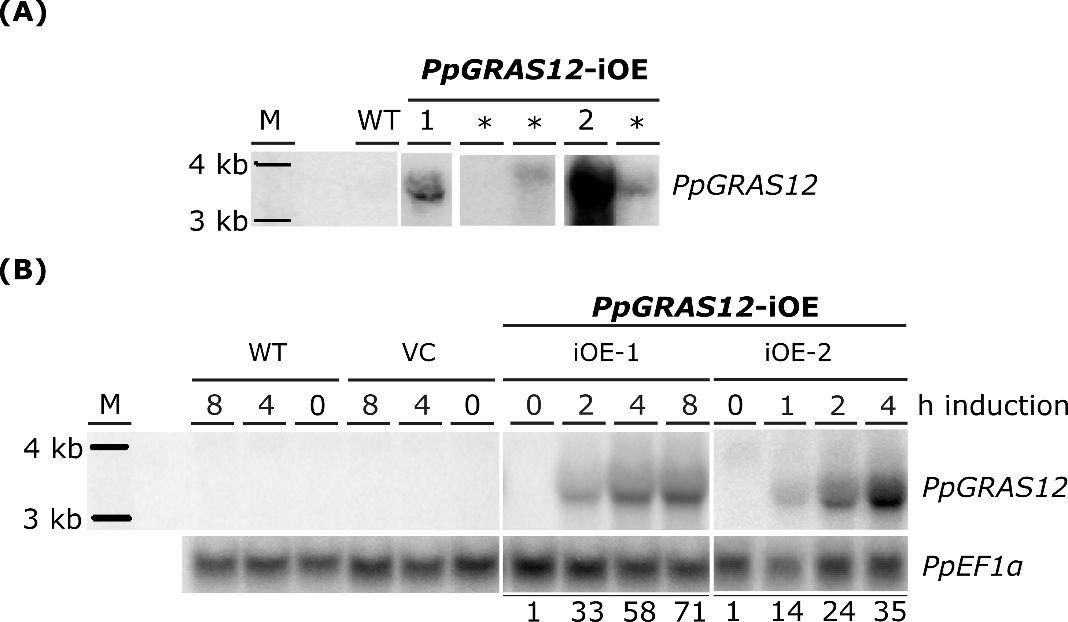


**Fig. S4** Validation of β-estradiol induced *PpGRAS12* expression in protonema tissues of *PpGRAS12*-iOE lines by Northern blots. **a** Expression of *PpGRAS12* 2 h after 1 μM β-estradiol induction in iOE lines as well as untreated WT. 5 μg total RNA were used for each sample. Lanes marked with asterisk shows iOE lines that were not selected for further analysis. **b** Time course analysis of *PpGRAS12* transcript quantities in iOE lines in comparison to WT and VC (vector control; empty GX8 plasmid). Normalized *PpGRAS12* expression levels (against *PpEF1α)* for iOE lines are shown under each lane. 8 μg total RNA were used for each sample. *PpEF1α*: RNA loading control for normalization. M: RNA marker.

**Table S1 List of primers used for the experiments**

| **Gene ID** | **Accession No. (V6.1 and V3.1)** | **Forward primers (5’ ⇨ 3’)** | **Reverse primers (5’ ⇨ 3’)** | **Amplicon**  **size (bp)** | **Application** |
| --- | --- | --- | --- | --- | --- |
| ***Ef1α*** | ***Pp1s7_445V6.1*** ***Pp3c2_6650V3.1*** | AGCGTGGTATCACAATTGAC | GATCGCTCGATCATGTTATC | 412 | Amplification of the elongation factor 1α from the cDNA |
| ***Ef1α*** | ***Pp1s7_445V6.1 Pp3c2_6650V3.1*** | AGCGTGGTATCACAATTGAC | GATCGCTCGATCATGTTATC | 660 | Amplification of the elongation factor 1α from the genomic DNA |
| ***Ef1α*** | ***Pp1s7_445V6.1 Pp3c2_6650V3.1*** | GTACCTCCCAGGCTGACTGC | GTGCTCACGGGTCTGTCCAT | 95 | qRT-PCR |
| ***C45*** | ***Pp1s107_181V6.1Pp3c13_2360V3.1*** | ACGCACCGGCATCGT | TGCTTGTTCATCACGACACCA | 90 | qRT-PCR |
| ***nptII*** | ***MK204379.1*** | gacaatgagttcgggtaatcccATCGGATCCTGTCAAACACTGA | GACAGGAGGCCCGATCTAGTAA | 1508 | Amplification of *nptII* from the PBSNNNEV vector |
| ***PpGRAS12*** | ***Pp1s205_1V6.1***  ***Pp3c12_10V3.1*** | ggg**GAGCTC**GAGCTCTCATTGCCGAGTACCG | ggg**GAATTC**GCAGATCCACGAGGACGCAGCC | 1482 | Generation of the 5´ flanking part of the PpGRAS12::GUS construct  **GAGCTC:** *Sac*I restriction site |
| ***GUS*** | ***-*** | ggg**GAATTC**ATGGTCCGGCCGGTAGAAACCC | ggg**GTCGAC**TCATTGTTTGCCTCCCTGCTGC | 1812 | Primers were used to amplify *GUS* coding sequence  **GAGCTC:** *Sac*I restriction site. **GAATTC:** *Eco*RI restriction site |
| ***PpGRAS12*** | ***Pp1s205_1V6.1***  ***Pp3c12_10V3.1*** | AgctcttcGGGCCAAGGGCAAAGGGAGA | ATCCTGCTACCTGTTGGCAG | 958 | Amplification of 5´UTR of *PpGRAS12* for generation of the ∆*PpGRAS12* mutants |
| ***Aph4*** | **-** | CCAACAGGTAGCAGGATAGGCTTTTCTCGACT | GCACCTACATTAAACTATCAATACACTCGACAGTTTTGA | 2174 | Amplification of hygromycin selection cassette for generation of the ∆*PpGRAS12* mutants |
| ***PpGRAS12*** | ***Pp1s205_1V6.1***  ***Pp3c12_10V3.1*** | TTGATAGTTTAATGTAGGTG | AgctcttcCATACTGACTAGCAACATAA | 904 | Amplification of 3´UTR of *PpGRAS12* for generation of the ∆*PpGRAS12* mutants |
| ***PpGRAS12*** | ***Pp1s205_1V6.1***  ***Pp3c12_10V3.1*** | CACCATGGTGATCACTGCAGGAAG | CTGCGTCCTCGTGGATCTGC | 2443 | Amplification of *GRAS* CDS for OE construct |
| ***PpGRAS12*** | ***Pp1s205_1V6.1***  ***Pp3c12_10V3.1*** | TGTCTTGGAATGCAGGGAGGATGTC | TGTCAACCTAACCGTGGGAACCGCCTTT | 721 | Amplification of a probe for Northern blot |
| ***PpGRAS12*** | ***Pp1s205_1V6.1***  ***Pp3c12_10V3.1*** | ggg**GTCGAC**TTGATAGTTTAATGTAGGTGCT | ggg**GGTACC**CATCAAAGTTTCCTTGTTGCAT | 1528 | Generation of the 3´ flanking part of the PpGRAS12::GUS construct |
| ***PpGRAS12*** | ***Pp1s205_1V6.1***  ***Pp3c12_10V3.1*** | GCTTTCTCAAAGAAATGCTCTCA | CGCCCTGATGCTCCATCACT | 2009 | Confirmation of the 5´ integration of the PpGRAS12::GUS lines |
| ***PpGRAS12*** | ***Pp1s205_1V6.1***  ***Pp3c12_10V3.1*** | ATGGTCCGGCCGGTAGAAACCC | AGAGAGCGTCATTTTATAGCTTAGCC | 1917 | Confirmation of the 3´ integration of the PpGRAS12::GUS lines |
| ***PpGRAS12*** | ***Pp1s205_1V6.1***  ***Pp3c12_10V3.1*** | TGTCACAGGATCGGGTCCTGCA | CACAATAGTCTAGAGAGCGT | 2863 | GRAS12::GUS-screen |
| ***PpGRAS12*** | ***Pp1s205_1V6.1***  ***Pp3c12_10V3.1*** | TGTCAACCTAACCGTGGGAACCGCCTTT | TGTCTTGGAATGCAGGGAGGATGTC | 700 | Confirmation of the loss of *PpGRAS12* transcript for screening of the ∆*PpGRAS12* mutants |
| ***PpGRAS12*** | ***Pp1s205_1V6.1***  ***Pp3c12_10V3.1*** | ggg**GTCGAC**ATGGTGATCACTGCAGGAAGTA | aaa**AGATCT**GCAGATCCACGAGGACGCAG | 2457 | Amplification of the full-length *PpGRAS12* coding sequence to generate the PpGRAS12::citrine construct.  **GTCGAC:** *Sal*I restriction site. **AGATCT:** *Bgl*II restriction site. |
| ***PpGRAS12*** | ***Pp1s205_1V6.1***  ***Pp3c12_10V3.1*** | TGTCTTGGAATGCAGGGAGGATGTC | TGTCAACCTAACCGTGGGAACCGCCTTT | 721 | Probe  analysis of the *PpGRAS12-*iOE lines via RNA gel blot |
| ***PpCLV1a*** | ***Pp1s5_68V6.1***  ***Pp3c13_13360V3.1*** | TGGTTTGTGTATGAGATGGTCGGA | TCGGCTGGAGGTGCAAAACGC | 127 | qRT-PCR |
| ***PpCLV1b*** | ***Pp1s14_447V6.1 Pp3c6_21940V3.1*** | GCTCCTACGGTTACATCGCGCC | CCCCGTCGCCAAACTCGCTC | 138 | qRT-PCR |
| ***PpCLE1*** | ***Pp1s87_125V6.1***  ***Pp3c7_11040V3.1*** | CCACGAACTCGAGCAAGC | CGTCGATCTTCGGGGAAT | 63 | qRT-PCR |
| ***PpCLE2*** | ***Pp1s86_83V6.1***  ***Pp3c1_13720V3.1*** | CAGCTTCAAAGGCGAGTGAT | GGCTTGGTCTGATCAGGAGT | 61 | qRT-PCR |
| ***PpCLE4*** | ***Pp1s6_223V6.1***  ***Pp3c26_11430V3.1*** | TCTGCTGGAGCTTTGCTATG | TCAGGAAGAGACCGGCTAGA | 73 | qRT-PCR |
| ***PpCLE5*** | ***Pp1s275_39V6.1***  ***Pp3c22_4590V3.1*** | GGACCCGATTTGGGATGT | TGGACATCCCCCACAGAC | 77 | qRT-PCR |
| ***PpCLE6*** | ***Pp1s292_57V6.1***  ***Pp3c19_6950V3.1*** | CGAATGTGGACTCGGTTGT | CATGAAATGCAACACCCTTCT | 60 | qRT-PCR |
| ***PpCLE7*** | ***Pp1s27_259V6.1***  ***Pp3c21_5600V3.1*** | AGCTTGAGTGCCGTGGTAGT | ACTTCCTGGGCTGTTCTGAG | 76 | qRT-PCR |
| ***PpAPB1*** | ***Pp3c15_24980V3.1*** | GACGGCAAGTCAAATGCAC | GACGTCTGCTGCTCTTCGTT | 60 | qRT-PCR |
| ***PpAPB4*** | ***Pp3c15_24790V3.1*** | GACGGCAAGTCAAATGCAC | GACGTCTGCTGCTCTTCGTT | 61 | qRT-PCR |
| ***PpNOG1*** | ***Pp3c1_11420V3.1*** | CGGAGCAGGGAAATCTCA | GATCAGCTTCCGAGTTAGATTGA | 72 | qRT-PCR |
| ***PpDEK1*** | ***Pp1s173_19V6.1***  ***Pp3c17_17550V3.1*** | CTGCCAACCCTTCAGACG | CTGCACTCAAAAACCAGCAA | 60 | qRT-PCR |
| ***PpRPK2*** | ***Pp1s311_57V6.1***  ***Pp31c7_5570V3.1*** | CATGCTGGATTTGTCCTTCA | CAAGTTCGCTAGCCCCTTC | 61 | qRT-PCR |
